# Antifungal Effect of Metabolites from a New Strain *Lactiplantibacillus Plantarum* LPP703 Isolated from Naturally Fermented Yak Yogurt

**DOI:** 10.3390/foods12010181

**Published:** 2023-01-01

**Authors:** Qian Peng, Jing Yang, Qiang Wang, Huayi Suo, Ahmed Mahmoud Hamdy, Jiajia Song

**Affiliations:** 1 College of Food Science, Southwest University, Chongqing 400715, China; 2 Chongqing Key Laboratory of Speciality Food Co-Built by Sichuan and Chongqing, Chongqing 400715, China; 3 Chongqing Agricultural Product Processing Technology Innovation Platform, Chongqing 400715, China; 4 Chongqing Engineering Research Center for Processing & Storage of Distinct Agricultural Products, Chongqing Technology and Business University, Chongqing 400067, China; 5 Dairy Science Department, Faculty of Agriculture, Assiut University, Assiut 71526, Egypt

**Keywords:** *Lactiplantibacillus plantarum*, metabolites, antifungal activity, biological control

## Abstract

The antifungal effect of metabolites produced by a new strain of *Lactiplantibacillus (Lpb.) plantarum* LPP703, isolated from naturally fermented yak yogurt, was investigated. The results showed that *Lpb. plantarum* LPP703 significantly inhibited four fungal species, including *Penicillium* sp., *Rhizopus delemar*, *Aspergillus flavus*, and *Aspergillus niger*. The metabolites produced after 20 h of *Lpb. plantarum* LPP703 fermentation showed the highest antifungal activity against *Penicillium* sp. Compared with the control group, the *Lpb. plantarum* LPP703 metabolites-treated *Penicillium* sp. spores were stained red by propidium iodide, indicating that the cell membrane of the fungal spores was damaged. Moreover, the antifungal effect of the *Lpb. plantarum* LPP703 metabolites on *Penicillium* sp. was not changed after heating or treatment with various proteases, but showed a sharp decrease when the pH value was regulated to 5.0 or above. The oleamide, trans-cinnamic acid, and citric acid were the three most abundant in the *Lpb. plantarum* LPP703 metabolites. Molecular docking predicated that the oleamide interacted with the active site of lanosterol 14-alpha-demethylase (CYP51, a crucial enzyme for fungal membrane integrity) through hydrogen bonds and had the lowest docking score, representing the strongest binding affinity to CYP51. Taken together, the metabolites from a new strain of *Lpb. plantarum*, LPP703, had potent antifungal activity against *Penicillium* sp., which might be associated with the damage of the active ingredient to fungal membrane integrity. This study indicated that *Lpb. plantarum* LPP703 and its metabolites might act as biological control agents to prevent fungal growth in the food industry.

## 1. Introduction

Fungi can contaminate different kinds of foods, such as milk, meat, fruits, vegetables, and cereals, resulting in the deterioration of these foods [1]. At present, food spoilage caused by fungal growth has become a major concern worldwide. In fact, between 25 and 40% of food commodities in several developing countries are lost due to fungal spoilage [2]. Besides causing considerable economic losses, fungal spoilage has a negative effect on public health and food security because of mycotoxin production. The consumption of foods contaminated with fungi may cause diverse and powerful toxic effects, including teratogenicity, carcinogenicity, and mutagenicity [3]. Although certain antifungal chemical compounds are applied in the control of fungal contamination, the development of fungal resistance and potential health risks limit the use of these chemicals [4]. Thus, it is essential to develop an effective and safe strategy to solve the issue of fungal spoilage.

During the past few years, biological preservation using protective microorganisms or their metabolites has received increasing attention. The lactic acid bacteria (LAB) have received particular attention due to their generally recognized as safe status. It has been reported that LAB have potent antifungal activity against many fungal genera [5,6,7]. Various bioactive metabolites (such as proteinaceous substances, cyclic dipeptides, organic acids, fatty acids, and hydrogen peroxide) generated by LAB are closely associated with the inhibitory effect of LAB on fungi [8,9,10]. The production of these antifungal compounds depends on the strain specificity of LAB [11]. 

LAB naturally living in extreme environmental conditions, such as fermented foods, may have special physiological characteristics in order to overcome the adverse conditions [12]. Traditional naturally fermented foods may be a rich source for obtaining LAB with antifungal activity. In a previous study, *Lactobacillus* (L.) *fermentum* SHY10, which effectively inhibited the growth of *Staphylococcus aureus* was isolated from Chinese pickles [13]. In this study, a new strain *Lactiplantibacillus (Lpb.) plantarum* LPP703 was isolated from naturally fermented yak yogurt, and its antifungal effect was hypothesized. Firstly, the antifungal effect of *Lpb. plantarum* LPP703 and its metabolites and the loss of fungal cell membrane integrity caused by the metabolites were assayed. Secondly, the effect of heat, protease hydrolysis, and pH on the antifungal activity of the metabolites was determined. Furthermore, the chemical composition of the metabolites was analyzed, and the antifungal substances were predicted by molecular docking with lanosterol 14-alpha-demethylase (CYP51), a crucial enzyme for fungal membrane integrity. The current research reports for the first time the antifungal effect of the metabolites from a new strain of *Lpb. plantarum*, LPP703.

## 2. Materials and Methods

### 2.1. Materials

Potato dextrose broth (PDB) medium, agarose, and agar were bought from Beijing Solarbio Technology Co. Ltd. (Beijing, China). The de Man, Rogosa, and Sharpe (MRS) medium was purchased from Beijing Land Bridge Technology Co., Ltd. (Beijing, China). The blood agar base was purchased from Beijing Aobo Star Biotechnology Co., Ltd. (Beijing, China), sterile defibrinated sheep blood was obtained from Nanjing Quanlong Biotechnology Co., Ltd. (Nanjing, China), and propidium iodide (PI) was purchased from Beyotime Institute of Biotechnology (Shanghai, China). Formic acid, acetonitrile, and isopropanol were purchased from Dongguan Sparta Chemical Co., Ltd. (Dongguan, China). 

### 2.2. Strains

*Penicillium* sp. (AF 93302), *Aspergillus flavus* (AF 93328), and *Aspergillus niger* (AF 91006) were from the China Center for Type Culture Collection (Wuhan, China). *Rhizopus delemar* was from contaminated Yongchuan bean paste (Chongqing, China). The culture medium of *Penicillium* sp., *Aspergillus flavus*, *Aspergillus niger*, and *Rhizopus delemar* was potato dextrose agar (PDA), and the culture temperature was 28 °C.

*Lpb. plantarum* LPP703 (GenBank accession number is OQ064461) was isolated from naturally fermented yak yogurt (Hongyuan County, Sichuan, China). In brief, the yogurt was sampled aseptically, which was serially diluted using 0.9% (*w*/*v*) sterile saline (starting from 10^−1^ to 10^−6^). These dilutions were swabbed on an MRS agar plate, followed by incubation at 37 °C for 48 h. A colony on the plate was selected and then streaked on an MRS agar plate until a pure culture was obtained. The purified isolate was mixed with 10% (*w*/*v*) skim milk and 0.1% (*w*/*v*) sodium glutamate, lyophilized, and then sealed in an evacuated ampoule. The obtained ampoule strain was stored at 4 °C.

### 2.3. Morphological Analysis, Identification and Safety Evaluation of Lpb. Plantarum LPP703

The *Lpb. plantarum* LPP703 was activated in MRS medium at 37 °C for 24 h. After two generations, the bacteria were collected, and then resuspended in sterile saline to prepare a bacterial suspension with an OD_600 nm_ value of 1.4 (bacterial cell numbers, 1.57 × 10^10^ cfu/mL). The bacterial culture was streaked on MRS agar plates, cultured at 37 °C for 24 h, and then the colony morphology was observed. The Gram staining of *Lpb. plantarum* LPP703 was carried out according to a standard procedure, as previously described [14]. The morphology of *Lpb. plantarum* LPP703 was observed by a Hitachi SU8010 microscope (Tokyo, Japan) according to the procedure described previously [15].

The 16S rDNA sequence analysis was performed to identify the *Lpb. plantarum LPP703* strain. The template DNA of the strain was extracted using the TIANGEN Genomic DNA kit (Beijing, China) and amplified by the polymerase chain reaction. The amplification products were separated by agarose gel electrophoresis on a 1.2% agarose gel, and then the 16S rDNA sequence analysis was performed by Beijing Genomics Institute (Shenzhen, China). The basic local alignment search tool and the MEGA 5.0 software were used to perform the homology analysis of the sequence and the construction of the phylogenetic tree [16,17].

The hemolytic activity of the *Lpb. plantarum* LPP703 strain was determined for safety assessment. The *Lpb. plantarum* LPP703 was streaked on the surface of blood agar plates containing 5% (*v*/*v*) sheep blood. After 24 h of culture at 37 °C, the zone of clearance around the colonies was measured to estimate the level of hemolysis. *Staphylococcus aureus* (No. 25923, ATCC, VA, USA) was used as a reference.

### 2.4. Determination of Antifungal Activity of Lpb. Plantarum LPP703 and Its Metabolites

The antifungal effect of *Lpb. plantarum* LPP703 was assayed by the overlay method, as described previously [18]. In brief, a 10 μL aliquot of *Lpb. plantarum* LPP703 cultures was spotted on MRS agar plates, following 48 h of incubation at 37 °C. Then, the plates were overlaid with PDA medium with mold spores (1 × 10^6^ spores/mL, spores were counted using a hemocytometer), followed by 48 h of incubation at 28 °C. The diameters of the inhibition haloes formed around the spots were measured.

The inhibitory activity of *Lpb. plantarum* LPP703-produced metabolites against fungi was also evaluated. The *Lpb. plantarum* LPP703 cultures were inoculated into MRS medium for 48 h of culture at 37 °C. The bacterial cultures were sampled every 4 h to determine the pH value and OD_600 nm_ value. The *Lpb. plantarum* LPP703 cultures were collected by centrifugation at different culture times, and then the supernatants were further filtered through a 0.2 μm membrane filter. The antifungal effect of the obtained cell-free supernatant (CFS) was assayed by microplate inhibition analysis [7]. Briefly, a 100 μL aliquot of *Lpb. plantarum* LPP703 CFS or MRS broth (as a control) was mixed with 100 μL PDB medium with mold spores (1 × 10^6^ spores/mL) in a 96-well plate. After 48 h of culture at 28 °C, the OD_600 nm_ value of each well was recorded. Results are expressed as the antifungal rate.

### 2.5. Determination of the Permeability of Cell Membrane

The cell membrane damage was further corroborated using PI staining according to a previously reported method [19] with minor modifications. Briefly, the spores of *Penicillium* sp. were treated with lyophilized *Lpb. plantarum* LPP703 CFS (final concentration, 62.5 mg/mL) for 48 h. Then, PI (1 μL/mL) was used to stain the spores for 20 min in darkness. Then, the spores were visualized under an Olympus BX53 fluorescence microscope (Olympus Corporation, Tokyo, Japan) after washing and centrifugation.

### 2.6. Antifungal Activity Stability of Metabolites from Lpb. Plantarum LPP703

The stability of the antifungal effect of metabolites from *Lpb. plantarum* LPP703 was investigated. To determine the heat sensitivity of the antifungal effect, aliquots of *Lpb. plantarum* LPP703 CFS were separately subjected to 60, 80, 100, and 121 °C for 2 h. The sensitivity of *Lpb. plantarum* LPP703 CFS to various kinds of proteases was investigated by incubating aliquots of the CFS separately with 1 mg/mL of pepsin, trypsin, protease K, and papain (BioFroxx, Einhausen, Germany). The hydrolysis reaction was performed under the optimal conditions for each protease. After 2 h, the hydrolysis was terminated by boiling at 100 °C for 5 min. The pH sensitivity was assessed by adjusting the pH value of aliquots of *Lpb. plantarum* LPP703 CFS to 2.0, 3.0, 3.5, 4.0, 4.5, 5.0, 5.5, 6.0, 6.5, and 7.0. The antifungal effect of all the treated *Lpb. plantarum* LPP703 CFS was assayed by microplate inhibition analysis, as described in Section 2.4 above.

### 2.7. Component Analysis of Metabolites from Lpb. Plantarum LPP703

The components of lyophilized *Lpb. plantarum* LPP703 CFS were analyzed by ultra-high-performance liquid chromatography using an Exion LC AD™ system (AB SCIEX, Framingham, MA, USA) coupled with an AB SCIEX Triple Time-of-Flight (TOF) 5600 plus System (AB SCIEX). Twenty milligrams of samples were dissolved in 400 μL of the methanol/water solvent mixture (4:1, *v*/*v*). After full extraction, the supernatant was obtained by centrifugation and used for liquid chromatography-mass spectrometry analysis using a 100 mm × 2.1 mm, 1.8 µm ACQUITY UPLC HSS T3 column (Waters Corporation, Milford, MA, USA). The injection volume was 10 μL, the flow rate was 0.4 mL/min, and the column temperature was maintained at 40 °C. The acquired LC-MS raw data were processed using the Progenesis QI Software (Waters Corporation).

### 2.8. Molecular Docking Prediction

The molecular operating environment (MOE) software v2018.01011 (Chemical Computing Group, Montreal, Canada) was used in this study. The three-dimensional (3D) structure of CYP51 was obtained from the RCSB Protein Data Bank (PDB ID 3LD6). The two-dimensional structures of three compounds, namely oleamide, trans-cinnamic acid, and citric acid, were obtained from PubChem and converted to 3D structures using the MOE software. The native binding site in the structure of CYP51 was set as the binding pocket for these three compounds. The QuickPrep module (implemented in MOE) was used for the optimization of the protonation state of CYP51 and the orientation of hydrogens. The protocol of induced fit docking was used for the docking workflow. The London dG and GBVI/WSA dG scores were used to rank all docked poses of molecules. The best-ranked pose was selected as the final binding mode, which was rendered by PyMOL (www.pymol.org (accessed on 29 November 2022)).

### 2.9. Statistical Analysis

All data are expressed as the mean ± standard deviation of three samples. Statistical analysis was performed with the one-way analysis of variance and Tukey′s test (*p* < 0.05, significantly different).

## 3. Results

### 3.1. Identification and Hemolytic Activity of Lpb. Plantarum LPP703 Strain

**Figure 1:**
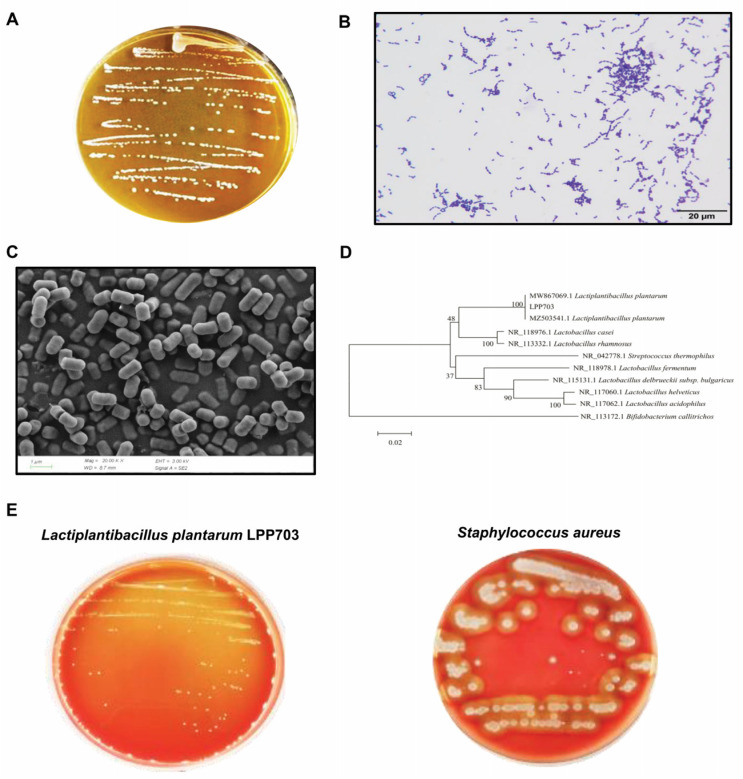
Morphology, identification, and safety analysis of the *Lactiplantibacillus (Lpb.) plantarum* LPP703 strain. (**A**) Colony morphology of *Lpb. plantarum* LPP703. (**B**) Gram staining of *Lpb. plantarum* LPP703. (**C**) Scanning electron microscopy image of *Lpb. plantarum* LPP703. (**D**) Neighbor-joining phylogenetic tree of *Lpb. plantarum* LPP703. (**E**) Hemolytic test of *Lpb. plantarum* LPP703.

The image of a representative culture plate in Figure 1A showed that the milky white colonies of the strain LPP703 had round morphology, a clean edge, and a smooth surface. After Gram staining, the colonies appeared purple in color (Figure 1B), indicating that the strain LPP703 was a Gram-positive bacterium. Additionally, as shown in Figure 1C, the strain LPP703 exhibited mostly a rod shape. The phylogenetic analysis showed that the strain LPP703 had 100% homology with two strains of *Lpb. plantarum* listed in the GenBank database (Figure 1D), which suggested that the LPP703 strain belonged to the *Lpb. plantarum* species. The safety of *Lpb. plantarum* LPP703 was further evaluated by the hemolytic test. The results revealed that *Lpb. plantarum* LPP703 did not show any hemolytic activity on blood agar plates (γ-hemolysis), while *Staphylococcus aureus* showed β-hemolytic activity. 

### 3.2. Antifungal Activity of Lpb. Plantarum LPP703 and Its Metabolites

**Figure 2:**
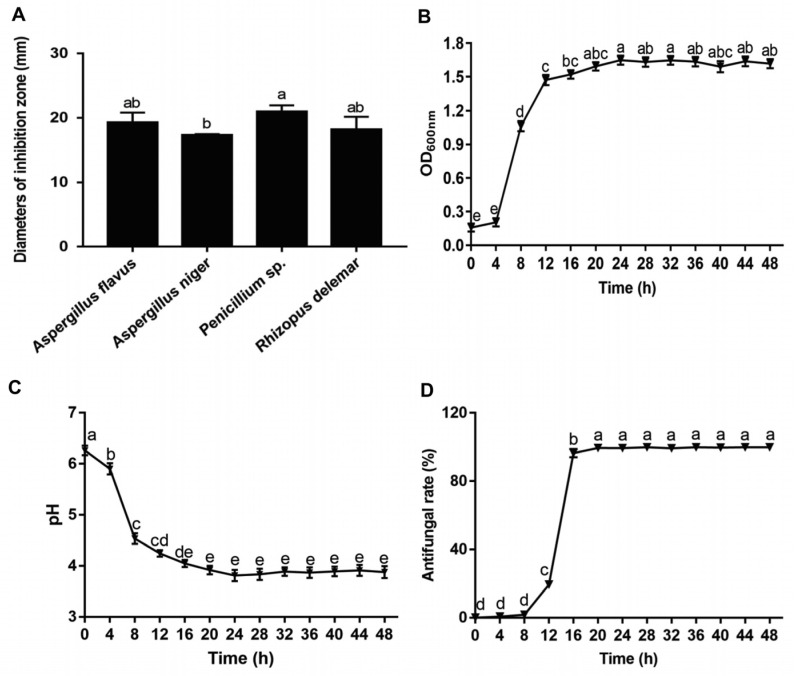
Antifungal activity of *Lactiplantibacillus (Lpb.)* plantarum LPP703 and its metabolites. (**A**) The inhibition effect of *Lpb. plantarum* LPP703 on *Aspergillus flavus*, *Aspergillus niger*, *Penicillium* sp., and *Rhizopus delemar*. (**B**) The growth curve of *Lpb. plantarum* LPP703. (**C**) The alteration in pH value during *Lpb. plantarum* LPP703 growth. (**D**) Inhibitory activity of *Lpb. plantarum* LPP703-produced metabolites against *Penicillium* sp. The one-way analysis of variance followed by Tukey’s test was used for statistical analysis. The different letters (a-e) denote significant differences (*p* < 0.05).

The determination of the inhibitory activity of *Lpb. plantarum* LPP703 against *Aspergillus flavus*, *Aspergillus niger*, *Penicillium* sp., and *Rhizopus delemar* showed that *Lpb. plantarum* LPP703 significantly inhibited the growth of these four fungi (Figure 2A). The diameter of the inhibition zone by *Lpb. plantarum* LPP703 against these four fungi ranged from 17.35 ± 0.13 to 21.00 ± 0.95 mm. The inhibitory activity of *Lpb. plantarum* LPP703 against *Aspergillus niger* was the weakest. Although there were no significant differences between the inhibitory activities of *Lpb. plantarum* LPP703 against *Aspergillus flavus*, *Penicillium* sp., and *Rhizopus delemar* (*p* > 0.05), the diameter of the inhibition zone of *Lpb. plantarum* LPP703 was maximal against *Penicillium* sp. Thus, *Penicillium* sp. was chosen as an indicator species for further investigation.

The antifungal effect of *Lpb. plantarum* LPP703 was further characterized by evaluating the antifungal effect of the metabolites produced during the first 48 h of culture. As shown in Figure 2B, the *Lpb. plantarum* LPP703 strain grew rapidly within 20 h, and then reached the stationary phase. Consistently, the pH value of the culture medium of the *Lpb. plantarum* LPP703 strain dropped dramatically from 0 to 20 h (Figure 2C). After 20 h, the pH was maintained at a stable acidic pH level. The CFS of the *Lpb. plantarum* LPP703 strain collected at 0, 4, and 8 h of fermentation time had no antifungal activity against *Penicillium* sp. (Figure 2D). However, from 12 to 20 h of culture, the antifungal activity of the collected *Lpb. plantarum* LPP703 CFS against *Penicillium* sp. showed a significant increase compared with that of the CFS obtained at the first 8 h of culture (*p* < 0.05). Afterwards, the antifungal activity of this CFS showed no significant alteration (*p* > 0.05). These results indicated that the metabolites were gradually accumulated with the extension of fermentation time, thus contributing to the antifungal activity of CFS.

### 3.3. Effect of Metabolites from Lpb. Plantarum LPP703 on Cell Membrane Integrity

**Figure 3:**
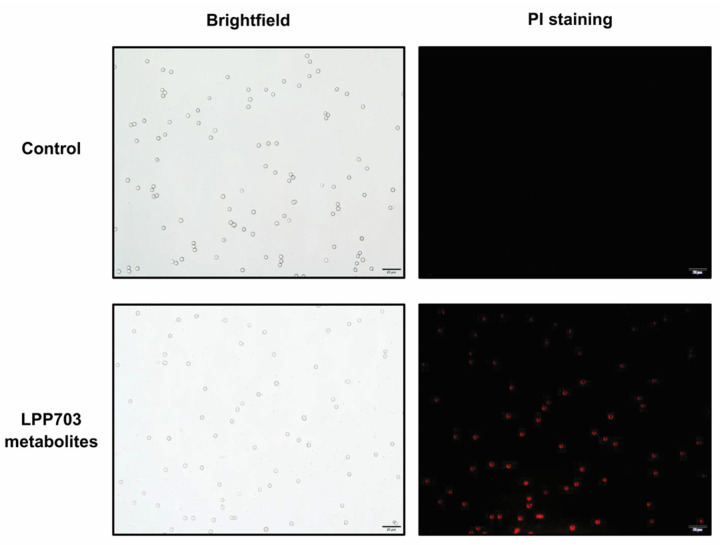
Effect of metabolites produced by the *Lactiplantibacillus plantarum* LPP703 strain on the cell membrane integrity of *Penicillium* sp. Scale bar, 20 μm.

The adverse effects of metabolites from *Lpb. plantarum* LPP703 on the fungal cell membrane were analyzed by fluorescence microscopy using PI staining. As shown in Figure 3, no fluorescence was observed in the untreated group, and the untreated *Penicillium* sp. spores grew well. In contrast, after treatment with metabolites from *Lpb. plantarum* LPP703, the bright red fluorescence was clearly visible. These results indicated that the metabolites from *Lpb. plantarum* LPP703 were able to damage the integrity of cell membranes, thereby contributing to the inhibition of fungi growth.

### 3.4. Effect of Heat, Enzymatic Hydrolysis, and pH on the Antifungal Activity of Metabolites from Lpb. Plantarum LPP703

**Figure 4:**
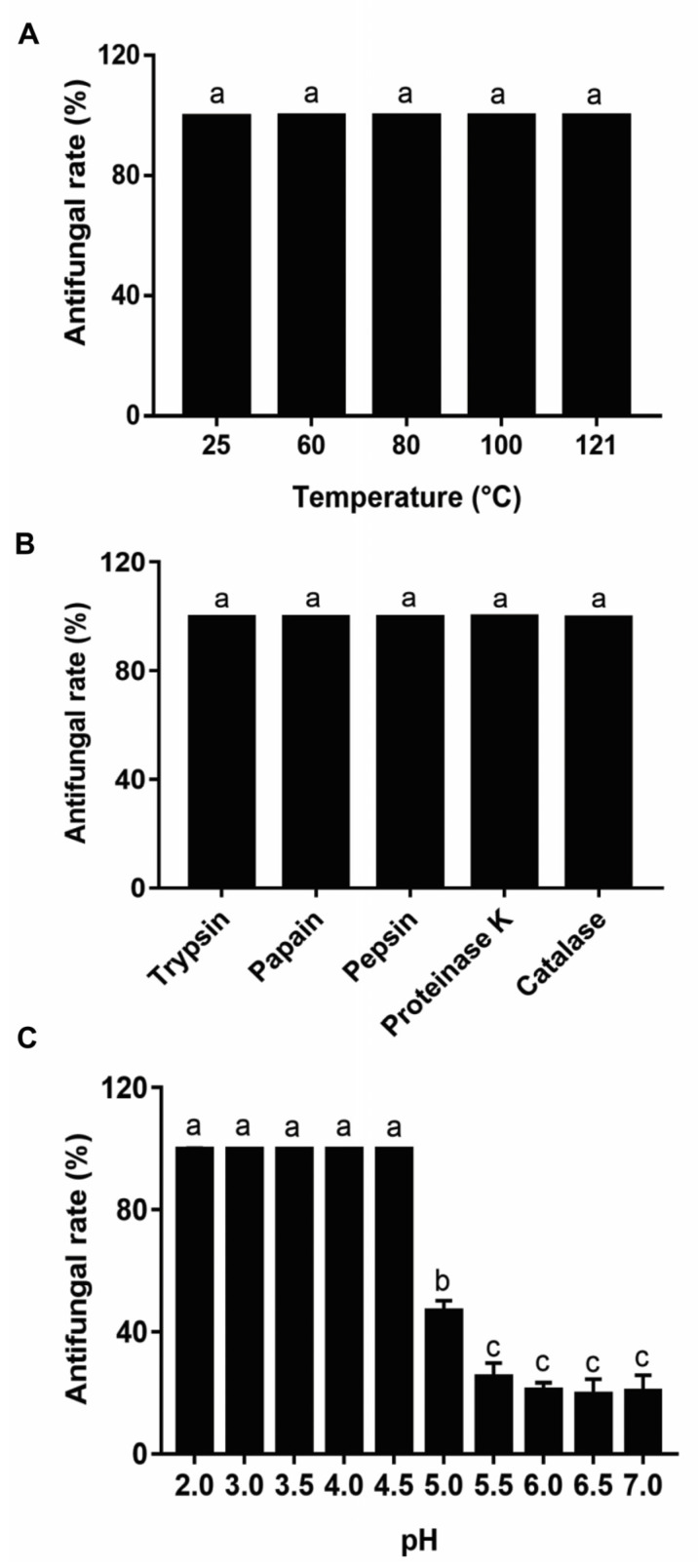
Antifungal activity stability of metabolites produced by the *Lactiplantibacillus plantarum* LPP703 strain. (**A**) Antifungal activity of heat-treated metabolites against *Penicillium* sp. (**B**) Antifungal activity of protease-treated metabolites against *Penicillium* sp. (**C**) Antifungal activity of metabolites after pH change against *Penicillium* sp. The one-way analysis of variance followed by Tukey’s test was used for statistical analysis. The different letters (a–c) denote significant differences (*p* < 0.05).

The effect of heat, enzymatic hydrolysis, and pH on the antifungal activity of the metabolites produced after 20 h of *Lpb. plantarum* LPP703 fermentation was investigated. As shown in Figure 4A, after the *Lpb. plantarum* LPP703 CFS was treated at different temperatures for 2 h, its antifungal effect on *Penicillium* sp. did not change significantly (*p* > 0.05). Moreover, the inhibitory activity of CFS against *Penicillium* sp. was not affected by treatment with trypsin, papain, pepsin, proteinase K, and catalase (Figure 4B). However, when the pH value of CFS was regulated to 5.0 or above, the antifungal effect of CFS showed a sharp decrease (Figure 4C, *p* < 0.05). At pH 5.0 and pH 7.0, the antifungal rates of CFS against *Penicillium* sp. deceased to 46.83 ± 3.46% and 20.54 ± 5.24%, respectively. These results suggested that the antifungal effect of *Lpb. plantarum* LPP703 CFS was stable after heat treatment and enzymatic hydrolysis, but sensitive to pH neutralization. 

### 3.5. Chemical Composition of Lpb. Plantarum LPP703 Metabolites and Molecular Docking against CYP51

The chemical composition of the CFS from *Lpb. plantarum* LPP703 culture was analyzed by LC-MS untargeted metabolomics. The results in Table 1 showed that fourteen compounds representing more than 1% of the total compounds were found in the *Lpb. plantarum* LPP703 CFS. Among these fourteen compounds, the amount of oleamide was the highest (40.90 ± 1.73%), followed by trans-cinnamic acid (5.31 ± 0.19%), and citric acid (4.18 ± 0.23%).

**Figure 5:**
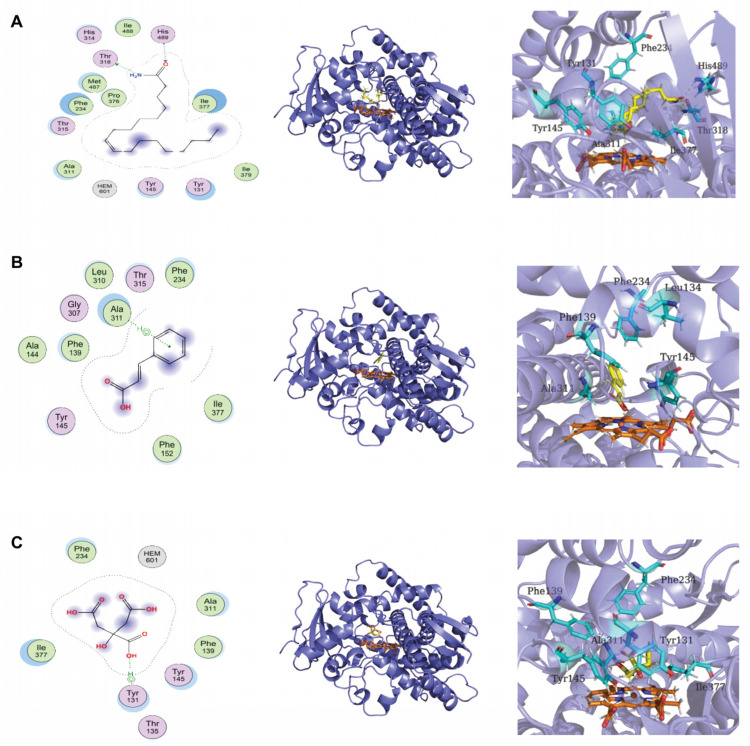
The molecular docking analysis of three metabolites produced by the *Lactiplantibacillus plantarum* LPP703 strain with lanosterol 14α-demethylase (CYP51). (**A**) The 2D and 3D binding modes of CYP51 with oleamide. (**B**) The 2D and 3D binding modes of CYP51 with trans-cinnamic acid. (**C**) The 2D and 3D binding modes of CYP51 with citric acid.

The analysis of molecular docking was used to determine the binding affinity of these three compounds to CYP51, an important enzyme for membrane integrity. The docking scores of oleamide, trans-cinnamic acid, and citric acid binding to CYP51 were −7.78, −4.66, and −5.10 kcal/mol, respectively (Table 2). The more negative docking score indicates stronger binding between CYP51 and compounds. Oleamide showed the strongest binding affinity to CYP51, and its amidogen and carboxide groups could interact with residues Thr318 and His489 of CYP51 through hydrogen bonds (Figure 5A). The 2D and 3D binding modes illustrated in Figure 5B,C revealed that the residues Ala311 and Tyr131 of CYP51 interacted with the benzene ring of trans-cinnamic acid and the hydroxide of citric acid through π-H interactions.

## 4. Discussion

LAB are considered to be the most promising candidates as fungal antagonists due to their GRAS status [20]. Fourteen Lactobacillus isolates from fermented Moroccan green olives show a high inhibitory effect on *Candida pelliculosa* [21]. The *L. plantarum* 4F strain is found to have a broad antifungal spectrum and inhibit eight fungal species, namely *Penicillium expansum*, *Aspergillus fumigatus*, *Aspergillus clavatus*, *Fusarium oxysporum*, *Penicillium brasilianum*, *Aspergillus niger*, *Candida albicans*, and *Aspergillus fisheri* [22]. The *L. plantarum* FJS003 strain significantly inhibits the growth and spore germination of *Aspergillus flavus* [15]. This study found that a new strain of *Lpb. plantarum* LPP703 showed a potent inhibitory effect on *Aspergillus flavus*, *Aspergillus niger*, *Penicillium* sp., and *Rhizopus delemar*, suggesting that the *Lpb. plantarum* LPP703 strain had the potential to be applied in fungal inhibition.

It is established that LAB can produce large amounts of metabolites in culture. The inhibitory activity of LAB against fungi is considered to be related to the active metabolites. The CFS of natural honey-derived *L. curvatus* HH strain inhibits the growth of *Candida glabrata*, *Candida parapsilosis*, and *Candida tropicalis*, which contributes to the inhibitory activity of *L. curvatus* HH against *Candida* spp. [23]. The CFS of the *L*. sp. RM1 strain from Egyptian traditional fermented milk exhibits broad inhibition on toxigenic fungi, including *Aspergillus carbonarious*, *Aspergillus parasiticus*, and *Aspergillus flavus* [24]. In the current study, the CFS obtained after 20 h of *Lpb. plantarum* LPP703 fermentation almost completely inhibited the growth of *Penicillium* sp. Further analysis of the action mode by which *Lpb. plantarum* LPP703 metabolites exerted their antifungal activity was performed by fluorescence microscopy using PI staining. PI, a fluorescent DNA intercalating dye, cannot enter into cells with an intact plasma membrane and is usually used as an indicator of disruption of plasma membranes [25,26]. The current study revealed that the treatment with metabolites produced by *Lpb. plantarum* LPP703 caused an increase in PI staining, which suggested that *Lpb. plantarum* LPP703 metabolites impaired the integrity of the cell membrane of fungi. Furthermore, the inhibitory activity of *Lpb. plantarum* LPP703 CFS against fungi was not affected by heat treatment, suggesting that the antifungal metabolites in the CFS of *Lpb. plantarum* LPP703 were highly resistant to heat. Treatment with proteases also did not change the inhibitory effect of *Lpb. plantarum* LPP703 CFS on fungi, indicating that the antifungal compounds in the *Lpb. plantarum* LPP703 CFS might not be proteinaceous in nature [27]. However, the antifungal activity of *Lpb. plantarum* LPP703 CFS against *Penicillium* sp. was reduced by pH neutralization, which was probably associated with the organic acids present in the CFS. When the pH is lower than the pKa of the organic acids, the non-dissociated form predominates and can cross the fungal cell membrane by passive diffusion, leading to the accumulation of organic acids in the cytoplasm and the inhibition of fungal growth. However, high pH causes organic acids to be dissociated, and these dissociated organic acids cannot readily penetrate through the cell membrane, decreasing the antifungal effect of organic acids [28,29]. Furthermore, the antifungal effect of some active compounds in the CFS is pH-dependent, and pH neutralization reduces their antifungal activity [30,31]. Consistent with the results obtained in the current study, the CFS from *L. buchneri* UTAD104 and *L. plantarum* UM55 retains the antifungal property after being autoclaved and hydrolyzed by proteases, but the antifungal effect of CFS is lost after pH neutralization [10].

Lactic acid (LA), an important metabolite produced by LAB, is identified as the main antifungal compound of *Lactobacillus casei* AST18 [31]. However, another study found that LA is not directly responsible for the antifungal effects of LAB strains on *Penicillium orylophilum* and *Aspergillus niger* [32]. LA alone has a weak inhibitory effect on *Penicillium corylophilum*, *Aspergillus niger*, and *Eurotium repens* [33]. The existing forms (dissociated and non-dissociated forms) of LA play a key role in exerting its antifungal activity. The pKa value of LA is 3.86, and a pH greater than 3.86 causes LA to be dissociated into a lactate anion. It is reported that 58% of the total LA is dissociated at pH 4, which is estimated to increase to 86% at pH 4.6 at 30 °C [34,35]. Some evidence has shown that the dissociation of organic acids limits their penetration into the fungal cells, thereby resulting in their low efficiency in inhibiting fungi [29,36]. In this study, the antifungal effect of *Lpb. plantarum* LPP703 CFS was not changed when the pH value was increased to 4.5, indicating that LA might not be primarily responsible for the antifungal activity of *Lpb. plantarum* LPP703 CFS. Thus, the composition of *Lpb. plantarum* LPP703 CFS using LC-MS-based untargeted metabolomics was further analyzed (Table 1). Oleamide, trans-cinnamic acid, and citric acid were the three most abundant metabolites. The level of oleamide is associated with the inhibition of the growth of *Aspergillus flavus* and *Fusarium oxysporum* [37]. Trans-cinnamic acid can be considered an effective antifungal organic acid against *Aspergillus flavus*, *Aspergillus parasiticus*, and *Colletotrichum* spp. [38,39]. Citric acid can change the permeability of the plasma membrane of fungi, contributing to its antifungal activity [40,41]. Although it was speculated that the antifungal effect of *Lpb. plantarum* LPP703 metabolites might be attributed to the synergistic action of various substances, the alone and combined antifungal activities of the identified compounds and their antifungal effect in foods need to be further investigated. In addition, molecular docking predicted that the three most abundant compounds could interact with the active site of Cyp51 by hydrogen bonding and π-H interactions. Cyp51 is critical for the synthesis of ergosterol, which is a core part of the fungal cell membrane, and inhibition of Cyp51 is considered to be an important target to limit fungal growth [42,43,44]. Whether the antifungal effect of *Lpb. plantarum* LPP703 metabolites was associated with the inhibition of Cyp51 needs to be verified in the future.

## 5. Conclusions

A new strain of *Lpb. plantarum*, LPP703, from naturally fermented yak yogurt, had a significant antifungal effect. The metabolites produced by *Lpb. plantarum* LPP703 damaged the cell membrane of *Penicillium* sp. spores, displaying potent antifungal activity against *Penicillium* sp. Oleamide, trans-cinnamic acid, and citric acid were abundant in the *Lpb. plantarum* LPP703 metabolites. They interacted with the active site of Cyp51 through hydrogen bonding and π-H interactions, which might contribute to the damage to the fungal cell membrane integrity caused by the *Lpb. plantarum* LPP703 metabolites. The current study provided promising biocontrol agents to combat fungal contamination in the food industry.

## Figures and Tables

**Table 1:** The chemical composition of metabolites produced by the *Lactiplantibacillus plantarum* LPP703 strain.

Compound	Chemical Formula	Retention Time (min)	Proportion (%)
Oleamide	C_18_H_35_NO	9.18	40.90 ± 1.73
Trans-Cinnamic acid	C_9_H_8_O_2_	2.07	5.31 ± 0.19
Citric acid	C_6_H_8_O_7_	1.04	4.18 ± 0.23
Linoleamide	C_18_H_33_NO	8.86	2.72 ± 0.83
2-Hydroxyphenethylamine	C_8_H_11_NO	2.07	2.72 ± 0.12
Omphalotin C	C_76_H_125_N_13_O_19_	3.97	2.29 ± 0.08
Desglucocheirotoxol	C_29_H_44_O_10_	2.97	2.16 ± 0.10
PS(MonoMe(11,5)/MonoMe(13,5))	C_50_H_86_NO_12_P	8.99	1.85 ± 0.11
CL(16:1(9Z)/22:6(4Z,7Z,10Z,13Z,16Z,19Z)/20:4(5Z,8Z,11Z,14Z)/20:4(5Z,8Z,11Z,14Z))	C_87_H_140_O_17_P_2_	3.97	1.59 ± 0.09
Neocasomorphin (1–5)	C_29_H_41_N_5_O_9_	2.92	1.28 ± 0.07
PE-NMe2(20:2(11Z,14Z)/22:4(7Z,10Z,13Z,16Z))	C_49_H_86_NO_8_P	8.79	1.22 ± 0.07
Ethyl 7-epi-12-hydroxyjasmonate glucoside	C_20_H_32_O_9_	2.38	1.15 ± 0.05
PE(22:2(13Z,16Z)/22:5(4Z,7Z,10Z,13Z,16Z))	C_49_H_84_NO_8_P	9.05	1.10 ± 0.07
PE(DiMe(13,5)/DiMe(13,5))	C_53_H_94_NO_10_P	8.72	1.06 ± 0.05

**Table 2:** The molecular docking scores of lanosterol 14α-demethylase (CYP51) with oleamide, trans-cinnamic acid, and citric acid.

Receptor	Ligand	Docking Score (Kcal/mol)
CYP51	Oleamide	−7.78
CYP51	Trans-Cinnamic acid	−4.66
CYP51	Citric acid	−5.10

## Data Availability

Not applicable.
